# Performance of the Edwards Sapien 3 bioprosthesis in atrioventricular valve position: a case series

**DOI:** 10.1093/ehjcr/ytae642

**Published:** 2024-12-05

**Authors:** Alejandra Garretano, Raul Abella, Joaquin Fernández Doblas, Antonio Pamiès Catalán, Paola Dolader Codina, Ferran Gran Ipiña, Laura Marfil Godoy, Pedro Betrián Blasco

**Affiliations:** Paediatric Interventional Cardiology Unit, Vall d’Hebron Hospital, Paseo de la Vall d'Hebron, 119-129, 08035 Barcelona, Spain; Pediatric Cardiac Surgery Department, Vall d’Hebron Hospital, Paseo de la Vall d'Hebron, 119-129, 08035 Barcelona, Spain; Pediatric Cardiac Surgery Department, Vall d’Hebron Hospital, Paseo de la Vall d'Hebron, 119-129, 08035 Barcelona, Spain; Pediatric Cardiac Surgery Department, Vall d’Hebron Hospital, Paseo de la Vall d'Hebron, 119-129, 08035 Barcelona, Spain; Pediatric Cardiology Department, Vall d’Hebron Hospital, Paseo de la Vall d'Hebron, 119-129, 08035 Barcelona, Spain; Pediatric Cardiology Department, Vall d’Hebron Hospital, Paseo de la Vall d'Hebron, 119-129, 08035 Barcelona, Spain; Pediatric Cardiology Unit, Arnau de Vilanova Hospital, Alcalde Rovira Roure avenue, 80, 25198 Lleida, Spain; Paediatric Interventional Cardiology Unit, Vall d’Hebron Hospital, Paseo de la Vall d'Hebron, 119-129, 08035 Barcelona, Spain

**Keywords:** Sapien 3, Tricuspid valve, Mitral valve, Congenital heart disease, Case report

## Abstract

**Background:**

The Edwards Sapien percutaneous valve (Edwards Lifesciences, Irvine, CA, USA) is a promising therapeutic option for congenital atrioventricular disease mostly because of the possibility to accommodate somatic growth with balloon dilatation.

**Case summary:**

This article reports the performance of the Edwards Sapien 3 valve in atrioventricular valve position in four paediatric patients.

**Discussion:**

Despite aggressive antiplatelet and anticoagulation strategies, most patients showed early bioprosthesis dysfunction, with increasing gradient not related with somatic growth. The decrease in leaflet motility in the absence of thrombosis or pannus could be associated with the low-velocity flow and low-pressure gradient that exists between the atria and ventricles in small children. It is well-known graft lifespan is usually shorter in small children, but we hypothesize the possibility of a second factor that valve design is intended to support higher flow-velocity patterns and can present an early failure in low-flow low-pressure situation. More studies are necessary to provide reliable evidence.

**Learning points**

- Atrioventricular valve disease in the paediatric population remains a therapeutic challenge due to the lack of availability of suitable implants, somatic growth, and the need of reinterventions.
- The third-generation Sapien prosthesis (S3 y Ultra) is a good option for aortic and pulmonary valve disease. However, atrioventricular valves work with lower patterns of flow and pressure gradients, and this could compromise the good functions of the Sapien prosthesis.

## Introduction

Atrioventricular (AV) valve disease in the paediatric population remains a therapeutic challenge due to the lack of availability of suitable implants and the implications of somatic growth. The Edwards Sapien percutaneous valve (Edwards Lifesciences, Irvine, CA, USA) is a balloon-expandable bioprosthesis approved for aortic and pulmonary valve position.^1^ The use of percutaneous bioprosthesis in the AV valve position is a promising therapeutic option like other authors reported mostly because of the possibility to accommodate somatic growth with balloon dilatation.^2^ We report our experience with the performance of the Edwards Sapien 3 valve in AV valve position in four paediatric patients.

## Summary figure

**Table:** 

**Patient 1**	
2 years old	Surgical implant of a Tricuspid SS3 23 mm
18 months later	Valve dysfunction, TTE: moderate PL, mild hypomotility of 1 leaflet, severe TR
4 years old	Percutaneous ‘valve-in-valve’ implant of a Sapien S3 23 mm
30 months later	Good valve function, TTE: mG < 5, without regurgitation but moderate–severe PL
**Patient 2**	
5 months old	Surgical implant of a Tricuspid SS3 23 mm
4 months later	Moderate stenosis of the prosthesis, TTE: mG 5–10 and moderate hypomotility of 1 leaflet. OAC treatment was commenced because of clinical suspicion of thrombosis.
30 months later	Progression of stenosis despite of OAC. TTE: mG > 10, severe TR, severe hypomotility of 3 leaflets.
**Patient 3**	
2 years and 8 months old	Surgical implant of a mitral SS3 23 mm
3 months later	Valve disfunction, TTE: mG > 10, without MR
2 years later	Balloon dilatation achieving a reduction in the mean gradient to 5 mmHg
6 months after dilatation	Progressive stenosis, TTE: mG > 10 and severe hypomotility of the 3 leaflets. She is awaiting further valve re-dilation.
**Patient 4**	
15 days old	Surgical implant of Tricuspid SS3 26 mm. Postoperative VA-ECMO
48 h later	Thrombosis of the bioprosthesis, anticoagulation and antiplatelet therapy was commenced
18 months later	Progressive stenosis, TTE: mG > 10, severe TR, severe hypomotility of 2 leaflet
1 year and 1 month old	Surgical ‘valve-in-valve’ implant of a new Tricuspid SU 26 mm
6 months later	Moderate stenosis, TTE: mG 5–10 with mild hypomotility of 1 leaflet

ECMO, extracorporeal membrane oxygenator; mG, mean gradient; MR, mitral regurgitation; OAC, oral anticoagulation; PL, paravalvular leak; SS, Sapien S3; SU, Sapien Ultra; TR, tricuspid regurgitation; VA: veno-arterial.

## Case reports

### Patient 1

A 2-year-old boy born with complex congenital heart disease and multiple previous interventions presented a severe tricuspid insufficiency and right heart failure. A 23 mm Edwards Sapien 3 was implanted surgically into tricuspid position, developing a severe regurgitation and paravalvular leak 3 months later due to infective endocarditis. At 4 years of age, a percutaneous ‘valve-in-valve’ procedure with a new valve implant (23 mm Edwards S3) and closure of a paravalvular leak with a device was performed without complications (*Table 1*). He was treated with acetylsalicylic acid (ASA) and clopidogrel afterwards. The follow-up echocardiograms show good function of the bioprosthesis 4 years after implantation with no stenosis or regurgitation; however, a moderate paravalvular leak has appeared, and the patient became symptomatic with multiple episodes of right heart failure (*Table 2*).

**Table 1:** Clinical and procedural characteristics

Case; age at implant (years)	Previous procedures	Interventional approach	Annulus	Prosthesis	Catheter dilation
1a; 2	Ross–Konno, TMP, RV-PA tube replacement	Surgical	20 × 20 mm (*z*-score +3)	Tricuspid SS3 23 mm	20 × 20 mm inflated to 16 atm (*z*-score +3)
1b; 4		Percutaneous ‘valve in valve’	Sapien S3 23 mm (*z*-score +1.7)	Tricuspid SS3 23 mm	24 × 20 mm inflated to 14 atm (*z*-score +3.6)
2; 0.6	Dammus–Kayle–Stansel	Surgical	18 × 20 mm (*z*-score +3.3)	Tricuspid SS3 23 mm	20 × 20 mm inflated to 16 atm (*z*-score +3.3)
3; 2.8	VSD closure, subaortic membrane resection, mitral repair	Surgical	19 × 21 mm (*z*-score +1.8)	Mitral SS3 23 mm	18 × 20 mm inflated to 16 atm (*z*-score +1.4)
4a; 15 days	Pulmonary balloon valvuloplasty	Surgical	22 × 22 (*z*-score +6.4)	Tricuspid SS3 26 mm	24 × 20 mm inflated to 14 atm (*z*-score +7.6)
4b; 1.1		Surgical ‘valve in valve’	Sapien Ultra 26 mm (*z*-score +4.1)	Tricuspid SU 26 mm	24 × 20 mm inflated to 14 atm (*z*-score +4.1)

atm, atmospheres; PA, pulmonary artery; RV, right ventricle; SS, Sapien S3; SU, Sapien Ultra; TMP, tricuspid mechanical prosthesis; VSD, ventricular septal defect.

**Table 2:** Data obtained through echo Doppler during follow-up

Case	Post-procedure TTE	Post-procedure *z*-score	Evolutive TTE^a^	Evolutive *z*-score	Treatment
1a	mG < 5, mild–moderate PL	3	**(24 m)** mG > 5, moderate PL, mild hypomotility of 1 leaflet, severe TR.	1.8	ASA 6 m followed by DAPT 1 year^b^
1b	mG < 5, mild PL	3.6	**(48 m)** mG < 5, moderate–severe PL.	0.81	DAPT 1 year followed by long term ASA
2	mG < 5, mild TR	3.3	**(30 m)** mG > 10, severe TR, severe hypomotility of 3 leaflet	1.01	DAPT 1 year followed by OCA
3	mG 5–10, without MR	1.4	**(30 m)** mG > 10, without MR, severe hypomotility of the 3 leaflets	1.03	DAPT 3 m followed by OAC
4	mG < 5, without TR/VA ECMO^c^	7.6	**(18 m)** mG > 10, severe TR, severe hypomotility of 2 leaflet	4.1	ASA + OAC
4b	mG < 5, without TR	4.1	**(6 m)** mG 5–10, without TR, mild hypomotility of 1 leaflet	3.4	ASA + OAC

ASA, acetylsalicylic acid; DAPT, double antiplatelet therapy; ECMO, extracorporeal membrane oxygenator; mG, mean gradient; MR, mitral regurgitation; OAC, oral anticoagulation; PL, paravalvular leak; TR, tricuspid regurgitation; VA, veno-arterial.

^a^The most altered individual values obtained during evolution are shown. However, significant valve changes in dysfunction begins earlier after implant (Case 1a: after 18 months; Case 1b: after 30 months; Case 2: after 6 months; Case 3: after 3 months; Case 4a: after 3 months; and Case 4b: after 3 months). Dosage detail: OAC: Case 2 was 0.1 mg/kg/d, Case 3 was 0.06 mg/kg/d, Case 4a was 0.2 mg/kg/d, and Case 4b was 0.06 mg/kg/d (adjusted for achieve a INR between 2.5 and 3.5). Clopidogrel: Cases 1a and 1b were 75 mg/d, Case 2 0.2 mg/kg/d, and Case 3 was 75 mg/d. ASA: 1–5 mg/kg/d.

^b^Valvular thrombosis.

^c^Withdrawal of clopidogrel because of bleeding. In bold: time at evolutive TTE.

### Patient 2

**Figure 1:**
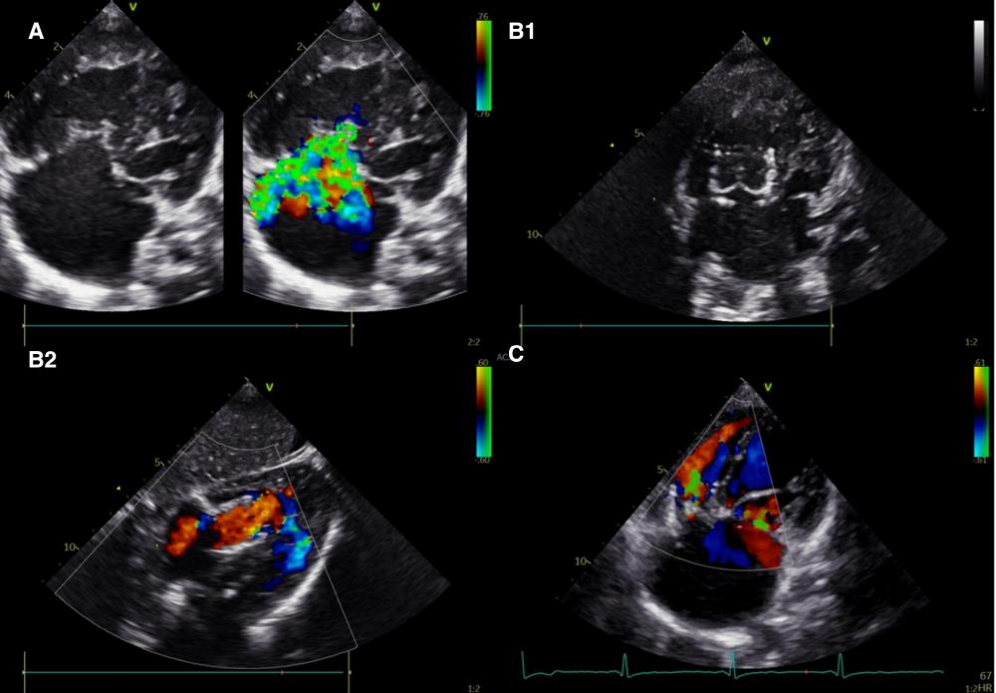
Evolutive functional changes in tricuspid valve (Case 2). (*A*) Transthoracic echocardiogram showing severe tricuspid regurgitation pre-surgery. (*B1*, *B2*) Transthoracic echocardiogram immediately after implant of the bioprosthesis. (*C*) Transthoracic echocardiogram 30 months after the implant with severe tricuspid regurgitation.

Patient 2 was a boy with univentricular congenital heart disease and severe tricuspid insufficiency. At 5 months of age, a 23 mm Edwards Sapien 3 was surgically implanted in the tricuspid valve position (*Table 1*). Initially, he was maintained on dual antiplatelet therapy with ASA and clopidogrel. Four months after implantation, the echocardiogram showed moderate hypomotility of the posterior valve leaflet and moderate valve stenosis (mean gradient 6–8 mmHg, annulus *z*-score +3). Due to clinical suspicion of thrombosis, anticoagulation treatment with acenocoumarol was commenced. Progression of valve stenosis occurred despite the absence of thrombi, observed as thickened leaflets with reduced motility on serial echocardiograms, resulting in severe stenosis and regurgitation 30 months after implantation (*Table 2*; *Figure 1*).

### Patient 3

Patient 3 was a girl with congenital mitral valve stenosis with previous surgical repair, who presented with residual severe mitral stenosis and regurgitation. At 2 years and 8 months of age, a 23 mm Edwards Sapien 3 valve was surgically implanted into mitral valve position (*Table 1*). She was initially treated with ASA and clopidogrel for 6 months, after which anticoagulation was started due to progressive stenosis. An increase in the mean pressure gradient to 15 mmHg with decreased leaflet motility, despite valve annulus *z*-score of 0, was observed at 8 months after implantation. At the age of 4 years and 8 months, 2 years after implantation, balloon dilation was performed with an Atlas Gold balloon catheter (Becton, Dickinson and Company, Franklin Lakes, USA) 22 mm × 20 mm at 14 atm, achieving a reduction in the mean gradient to 5 mmHg. The decision to perform a balloon valvuloplasty was based on the small size of the prosthesis to perform a ‘valve-in-valve’ procedure. She continued on anticoagulation therapy. The mean gradient progressively increased during follow-up, reaching 12 mmHg 2 years after the dilation of the bioprosthesis and with episodes of pulmonary oedema. With a current annulus *z*-score of −1.1 when adjusted for growth, she is awaiting further valve re-dilation (*Table 2*).

### Patient 4

A boy born with Ebstein's anomaly with severe tricuspid insufficiency, for which a 26 mm Edwards Sapien 3 was implanted using a surgical approach at the age of 15 days. He required postoperative mechanical support with extracorporeal membrane oxygenation. As anticoagulation was omitted due to bleeding, he developed thrombosis of the bioprosthesis 48 h after, which partially resolved with anticoagulation and antiplatelet therapy. One year and 10 months after the initial procedure, a new 26 mm Edwards Sapien Ultra valve was implanted surgically (*Table 1*). Two months later, despite anticoagulation and antiplatelet therapy (ASA), bioprosthesis dysfunction was observed with a mean gradient of 7 mmHg and reduced leaflets motility without evidence of thrombi (annulus *z*-score +3.6), which persists during the follow-up 6 months later (*Table 2*). The patient was asymptomatic and stable, waiting for the optimal timing to intervene.

## Discussion

The present case series reports the performance of the Edwards Sapien 3 bioprosthesis in AV valve position in four paediatric patients during short- and medium-term follow-ups.

Since Edwards Sapien valves have demonstrated excellent results in the percutaneous treatment of aortic valve stenosis in adult patients, some groups have extrapolated their experience, applying it to the treatment of native pulmonary valve dysfunction, right ventricle to pulmonary valve conduit failure, and degenerated pulmonary bioprostheses.^3,4^ Other dedicated bioprostheses, such as Melody valve (Medtronic; Minnesota, USA), designed for the percutaneous implantation into the right ventricular outflow tract, have previously been utilized for implantation into the AV valve position, mainly mitral, with good results.^5,6^ The current third-generation Edwards devices, the Edwards Sapien 3 and Sapien 3 Ultra, are balloon-expandable, bioprosthetic valves that offer the possibility of further dilation, thus increasing the inflow area, an essential characteristic in the paediatric population. The drawback of this treatment option is the longevity of the bioprosthesis. However, in recent years, due to technical improvements in design and materials, new evidence has emerged supporting their durability. An international registry reports that in short and medium term, the Sapien 3 valve in the pulmonary position performed well with a 6-year cumulative incidence of valve replacement of 8%, valve thrombosis of 0.7%, and infective endocarditis of 3.8%.^7^

In our case series, early dysfunction of the Sapien 3 bioprosthesis was observed. Despite aggressive antiplatelet and anticoagulation strategies, most patients showed early bioprosthesis dysfunction, with increasing gradient due to failure to accommodate for somatic growth (*Table 2*). One of the main concerns when performing valve replacement in paediatric patients is the exponential growth of the native valve annulus that occurs in the early stages. In this regard, the use of prostheses with fixed diameters poses significant limitations. The selection of the bioprosthesis in our patients was made according to the size of the annulus measured by echocardiography and correlated with the calculated *z*-score. In addition, a decrease in the motility of the valve leaflets was noted during the follow-up. The Edwards Sapien bioprosthesis was designed for the aortic valve replacement and later also approved for the pulmonary implantation. Both positions share significantly higher velocity flow patterns compared with AV valves. The decrease in leaflet motility in the absence of thrombosis or pannus formation could be associated with the low-velocity flow and low-pressure gradient that exists between the atria and ventricles, causing valve dysfunction. While in Case 4, the intracardiac volume depletion due to mechanical assistance, in combination with the absence of anticoagulation, could have promoted valve thrombosis, the second valve implant also developed early dysfunction.

As mentioned, the size selection of the bioprosthesis poses a significant decision dilemma. In Case 1, where the dimensions of the second valve implant fit more closely with the size of the child and the annulus, the performance during the follow-up was more favourable. However, although the performance may not be optimal in the short term with significant valve oversizing, in the long term, it provides an important advantage by allowing a greater number of implants using the ‘valve-in-valve’ technique as required.

With regard to pharmacological treatment, the initial regimen in most cases consisted of dual antiplatelet therapy. In Patient 4, anticoagulation was started early due to valve thrombosis. As shown in *Table 2*, the modifications in treatment responded to the observed haemodynamic changes of the prostheses and not the other way around.

Although the Edwards Sapien 3 bioprosthesis is an excellent therapeutic option in patients with aortic or pulmonary valve disease, the results in paediatric patients in the AV valve position are suboptimal. We hypothesize that the underlying factor associated with early valve deterioration could be its design to support higher flow-velocity patterns. It would be interesting to evaluate whether changes in the design of the prosthesis, for example the creation of individualized models through 3D printing, could improve its haemodynamic profile in this scenario.

## Conclusions

The Edwards Sapien 3 bioprosthesis is an excellent therapeutic option in patients with aortic or pulmonary valve disease; early dysfunction is observed in paediatric patients in AV position. More studies are necessary to provide reliable evidence of the performance of the third-generation Edwards Sapien bioprosthesis implanted in AV valve position in paediatric population.

## Data Availability

All available data are in the main manuscript.

## Lead author biography

Alejandra Garretano works as an interventional adult cardiologist in Uruguay. She run the Cardiology Residency in general Cardiology at the University Cardiovascular Center (Hospital de Clínicas Dr. Manuel Quintela, Facultad de Medicina, Universidad de la República). She is actually interested in structural interventions in CHD and Pulmonary Hypertension. During 2023 she finished a minifellowship in Vall d'Hebron Hospital (Barcelona, Spain) under the supervision of Pedro Betrian Blasco MD, PhD.

**Consent:** Parents signed written informed consent regarding publishing patient data in compliance with the COPE guidelines.

**Funding:** None declared.

## References

[1] Boone RH , Webb JG, Horlick E, Benson L, Cao QL, Nadeem N, et al Transcatheter pulmonary valve implantation using the Edwards SAPIEN™ transcatheter heart valve. Catheter Cardiovasc Interv 2010;75:286–294. 19924775 10.1002/ccd.22250

[2] Kahl BS , Michel-Behnke I, Zimpfer D. Double atrioventricular valve replacement using Melody™ transcatheter valves in an infant with unbalanced atrioventricular septal defect: a case report. Eur Heart J Case Rep 2020;4:1–6. 10.1093/ehjcr/ytaa174 PMC7501891 32974435

[3] Kenny D , Rhodes JF, Fleming GA, Kar S, Zahn EM, Vincent J, et al 3-year outcomes of the Edwards SAPIEN transcatheter heart valve for conduit failure in the pulmonary position from the COMPASSION multicenter clinical trial. JACC Cardiovasc Interv 2018;11:1920–1929. 30286853 10.1016/j.jcin.2018.06.001

[4] Abdullah I , Ramirez FB, McElhinney DB, Lock JE, Del Nido PJ, Emani S. Modification of a stented bovine jugular vein conduit (melody valve) for surgical mitral valve replacement. Ann Thorac Surg 2012;94:e97–e98. 23006723 10.1016/j.athoracsur.2012.02.101

[5] Quiñonez LG , Breitbart R, Tworetsky W, Lock JE, Marshall AC, Emani SM. Stented bovine jugular vein graft (Melody valve) for surgical mitral valve replacement in infants and children. J Thorac Cardiovasc Surg 2014;148:1443–1449. 24332108 10.1016/j.jtcvs.2013.10.059

[6] Hofmann M , Dave H, Hübler M, Kretschmar O. Simplified surgical-hybrid Melody® valve implantation for paediatric mitral valve disease. Eur J Cardiothorac Surg 2015;47:926–928. 25015952 10.1093/ejcts/ezu275

[7] Hascoët S , Bentham JR, Giugno L, Betrián-Blasco P, Kempny A, Houeijeh A, et al Outcomes of transcatheter pulmonary SAPIEN 3 valve implantation: an international registry. Eur Heart J 2024;45:198–210. 37874971 10.1093/eurheartj/ehad663

